# An eye tracking investigation of attention mechanism in driving behavior under emotional issues and cognitive load

**DOI:** 10.1038/s41598-023-43693-8

**Published:** 2023-10-08

**Authors:** Quan Wang, Feiyu Zhu, Ruochen Dang, Xiaojie Wei, Gongen Han, Jinhua Huang, Bingliang Hu

**Affiliations:** 1 Key Laboratory of Spectral Imaging Technology, Xi’an Institute of Optics and Precision Mechanics (XIOPM), Chinese Academy of Sciences, Xi’an, 710000 China; 2 University of Chinese Academy of Sciences, Beijing, China; 3 Key Laboratory of Biomedical Spectroscopy of Xi’an, Xi’an Institute of Optics and Precision Mechanics (XIOPM), Chinese Academy of Sciences, Xi’an, China; 4 Xi’an GaoXin No.1 High School, Xi’an, China; 5 Department of Pediatrics, The Third Affiliated Hospital of Anhui Medical University, Hefei, China

**Keywords:** Neuroscience, Psychology

## Abstract

Emotions have specific effects on behavior. At present, studies are increasingly interested in how emotions affect driving behavior. We designed the experiment by combing driving tasks and eye tracking. DSM-V assessment scale was applied to evaluate the depression and manic for participants. In order to explore the dual impacts of emotional issues and cognitive load on attention mechanism, we defined the safety-related region as the area of interest (AOI) and quantified the concentration of eye tracking data. Participants with depression issues had lower AOI sample percentage and shorter AOI fixation duration under no external cognitive load. During our experiment, the depression group had the lowest accuracy in arithmetic quiz. Additionally, we used full connected network to detect the depression group from the control group, reached 83.33%. Our experiment supported that depression have negative influences on driving behavior. Participants with depression issues reduced attention to the safety-related region under no external cognitive load, they were more prone to have difficulties in multitasking when faced with high cognitive load. Besides, participants tended to reallocate more attention resources to the central area under high cognitive load, a phenomenon we called "visual centralization" in driving behavior.

## Introduction

Attention is the direction of mental activity towards things that are meaningful to oneself, which affects cognitive and emotional functions^1^. The psychological enterprise revolves around attention, as Titchener noted more than a century ago. At the brain level, neuroscientists have categorized attention into three types: selective attention, executive attention, and vigilance^2^. In recent years, attention has become one of the fastest growing fields in cognitive psychology^3^. Many other issues in neuroscience and psychology have been investigated in combination with it, including consciousness, vigilance, saliency, executive control, and learning^4^. When it comes to driving behavior, we need to allocate our attention resources to manipulation, adjust and plan at the same time for safety^5^.

### Relate work

Driving behavior is a multidimensional issue, influenced by many factors, including driving attitude, driving fatigue level, emotion, cognitive load^6^. The motivation behind the driving behavior is determined by the attitude. Driving attitude converts the acquired knowledge into actions, which can greatly affect our driving safety. Driving fatigue level is another important factor affecting driving behavior. Lal and Craig^7^ studied the influence of driving fatigue level and pointed out that anxiety, personality and temperament can lead to driving fatigue.

At present, psychologists are increasingly interested in how emotions affect the driving behavior^8–11^. Anger have been related to crash involvement. Abdu et al.^8^ used a driving simulator to explore the relationship between anger and driving behavior, they found that angry drivers ran red lights more often, which increase the probability of traffic accidents. Sullman^9^ investigated the types of situations that cause drivers to become angry while driving, they found that in all possible anger-inducing situations, drivers who tended to be younger, less experienced, and preferred faster speeds were more likely to report higher levels of driving anger. Zimasa et al.^10^ used the mind wandering theory to explore changes in the driver's following behavior and gaze patterns when affected by different emotions. The results show that negative emotions lead to the most dangerous driving. According to Matthews^11^, drivers with strong anger qualities displayed more situational rage and then adopted riskier and more aggressive driving styles. Most previous studies focused on the effect of emotions like anger on driving behavior^12^, few research pay attention to the emotional issues. Cognitive deficiencies associated with emotional issues might affect mnemonic function and attention mechanism^13,14^. Beirness explored high level of depression place predisposes individuals them at a higher risk of crash involvement^15^.

The relationship between cognitive load and attention mechanism in driving behavior has been extensively studied based on eye tracking. On account of the robust influence of emotional issues on attention^16–19^. Edquist et al.^20^ examined the potential impact of billboards in roads on driving behavior and found that older and less experienced drivers were more susceptible to interference. They reported that the presence of billboards altered the pattern of the driver's visual attention and lengthened the amount of time required for the driver to react to traffic signs. Engström et al.^21^ investigated the effects of cognitive demands on driving performance and driver status, and their findings indicated cognitive load did not affect speed and leads to increased attention at the center of road. Desmet and Diependaele^22^ found that cell phone distractions could enhance the cognitive load of drivers, which is common risk factors for driving safety. Additionally, hands-free phones have been found to have a protective effect on the attention mechanism during driving^23,24^.

### Our work

As discussed above, previous studies have shown that emotions have specific effects on the attention mechanism of driving behavior, while few of them focused on the effect of emotional issues like depression or mania. Besides, the reciprocal influence between cognitive load and attention mechanism is of great interest^25,26^. Many previous studies have extensively explored the effects of cognitive load or emotional issues on the attention mechanism of driving behavior^8–15^. Furthermore, several studies have examined the combined influence of these factors^27,28^. In alignment with research integrating cognitive load and emotional issues, our study specifically aims to elucidate the dual effect on the attention mechanism of driving behavior.

Hands-free phone is the potential factor that increases the level of cognitive load, resulting in driving distractions. In our experiment, we set two levels of cognitive load, including the scene with no external cognitive load and the scene with arithmetic quiz answering. They are used to explore the interplay between cognitive load and attention mechanism in driving behavior.

Based on the purpose of our experiment, we take driving age into account, which is factor relevant in driving behavior. Driver age is an important factor affecting driving behavior^29^, however, there have been few studies involving adolescents. The initial age of driving begins to decline, presenting a trend of younger development^30^. There is a growing body of research analyzing the driving behavior of teenagers over the age of 16^31–35^. We focused on 16-year-old adolescents since research shows that they are more prone to traffic accidents due to their limited risk perception, emotional vulnerability, and inability to concentrate for extended periods while driving^36–38^. Shope et al.^31^ devised prediction models that used data from 794 individuals aged 16–21 to predict subsequent first-year driving collisions and infractions. Hartos et al.^33^ examined the driving behavior of 658 parents and their 16-year-old adolescents, finding that intended driving limits were more likely in cases where parents reported high levels of parental monitoring and risk perception. As the group about to get driving license, adolescents will be involved in transportation in the future. Besides, frustrations in their growth are prone to causing emotional issues^39^. Emotional issue is the leading cause of disability in the world, accounting for almost half^40,41^, which significant importance should be attached to it.

In our research, we design a driving-related experiment to investigate the dual effect of cognitive load and emotional issues on attention mechanisms of driving behavior. Specifically, we evaluate participants' emotional states using the DSM-V assessment scale, categorizing them into the control group, the depression group, and the mania group. The experiment utilizes eye tracking technology and incorporates two levels of cognitive load, distinguished by the presence or absence of external voice interference.

## Methods

### Participants

As to reduce gender differences, the selected participants were 52 male students ranging from 16 to 17 years old (M_age_ = 16.4, SD_age_ = 0.48) in high school. We take gaze capture as eye-tracking criteria that whose gaze capture less than 25% were excluded. DSM-V assessment scale was taken as the criteria to assess emotional state of adolescents in the experiment. Based on the result of DSM-V assessment scale, adolescents were divided into depression group, manic group, and control group. Demographic information about participants is displayed in Table 1, the mean and standard deviation are showed in it. This study was performed in line with the principles of the Declaration of Helsinki. Approval was granted by the Third Affiliated Hospital Ethics Committee of Anhui Medical University (Reference Number: 2020-020-01), Hefei, China. Written informed consent was obtained from all participants and their parents.

**Table 1:** Participants.

	Control	Manic	Depression
Participants	19	22	11
Age	16.38 ± 0.35	16.56 ± 0.43	16.28 ± 0.61
Depression-child age 11–17 scale	23.79 ± 4.38	25.68 ± 4.57	34.18 ± 2.04
The Altman self-rating manic scale	3.16 ± 1.89	9.27 ± 2.59	2.27 ± 1.90

### DSM-V assessment scale

The Diagnostic and Statistical Manual of Mental Disorders (DSM) is commonly regarded as the gold standard manual in assessing the psychiatric diseases^42^. The Chinese version of the DSM-V assessment scale is translated by Professor Jianping Wang from Beijing Normal University and Associate Professor Jie Zhong from Peking University. We have been authorized by Professor Jianping Wang to use the Chinese version of DSM-V assessment scale. We used three emotional assessment scales including level 1-Self-Rating Symptom Scale, level 2-Depression-Child Age 11–17 Scale and level 2-Altman Self-Rating Manic Scale (ASRM), they are all suitable for adolescents aged from 11 to 17 years.

Depression-Child Age 11–17 Scale has 14 questions that are used to assess the severity of depression or depressive symptoms in the past 14 days. All questions were rated on a five-point scale (1 = never, 5 = almost always), with scores ranging from 14 to 70, with higher scores indicating higher levels of depression. In this study, a depression scale score greater than 31 was selected as the criteria for the depression group. The ASRM is a five-item scale that evaluates whether a child or adolescent has had symptoms of manic disorder in the past seven days. All questions were rated on a five-point scale from 0 to 20 (0 = never, 4 = almost always), with the higher scores, the more severe the manic symptoms. In this experiment, a score of more than 5 on the scale was selected as the criterion for the manic group.

According to the scale scores, we divided the 52 adolescents into three groups, the control group, the depression group, and the mania group. 19 adolescents with no symptoms in both level 2 emotional assessment scale was taken as the control group, accounting for 36.54%. 22 adolescents were in the mania group, accounting for 42.31%, the other 11 adolescents in the depression group, accounting for 21.15%. It can be concluded that the proportion of the manic group was the highest, which was 6% higher than that of the control group and twice the proportion of the depression group.

### Experiment

**Figure 1:**
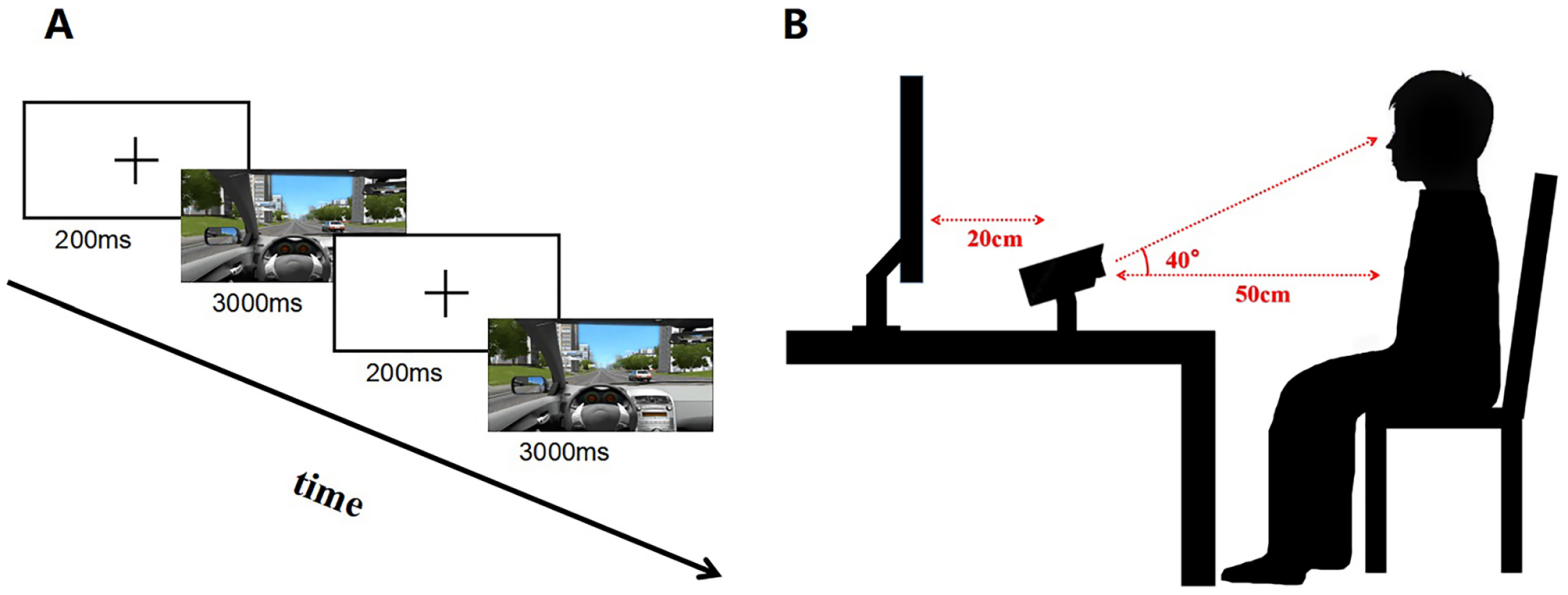
Schematic diagram of the experiment paradigm and the layout of the experimental scene.

City Car Driving presents the first-person perspective inside the car and provides real-time driving environment. We recorded 20 driving video clips from it and 10 question-and-answer audios by ourselves, each video clip or audio was 30 s. The video clips with arithmetic questions and the video clips without arithmetic questions accounted for 50% respectively. The audio consists of three arithmetic questions (e.g., 30 + 12 = ?), each question will be asked in the first 5 s and the participants need to answer in the next 5 s. Arithmetic task is a cognitive experiment to explore the cognitive load level or stress condition of participants, which has been widely used in psychological research^43,44^. Arithmetic tasks with different difficulty correspond to different levels of cognitive load. In our experiment, we divided the cognitive load level into high cognitive load and no external cognitive load according to whether the trials were synchronized with arithmetic task. We played two video clips with audio and two video clips without audio in an intersecting order, the interval between two videos was set to 2 s, as in Fig. 1A. The total duration of the experiment was about 11 min.

### Procedure

We complete synchronous playback of video clips with audio and set the sequence of videos with Psychopy. The desktop eye tracking instrument EyeCatch Bar is developed by Dongguan Dongquan Technology Co., Ltd, it can be calibrated at nine points with a sampling rate of 41 Hz and an accuracy of 1.25 degree. We invited one participant into the room at a time, the experimental environment was shown in Fig. 1B. A 23.8-inch LCD screen and the eye tracker were placed on the table, with 20 cm between them. During the experiment, the participants would fill out three DSM-V assessment scales first, then the eye tracker calibration was carried out. It would not pass until the mean error angle of the calibration was less than 1.25 degrees. Participants were asked to watch the video from the view of participating in traffic. At the same time, they have to answer the arithmetic quiz from external voice.

### Data analyzes

**Figure 2:**
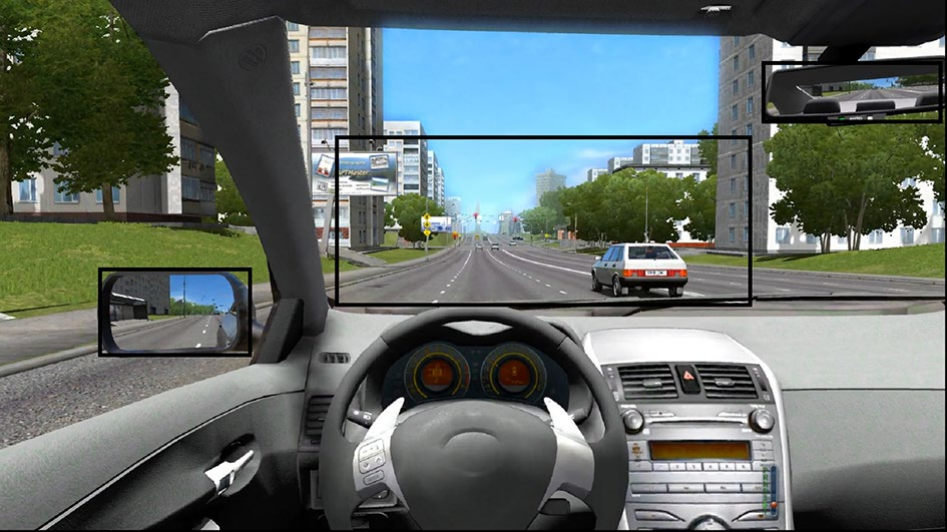
Simulated driving platform and AOI area set.

We defined area of interest (AOI) to measure the attention mechanism of participants, including the side rearview mirror, the rearview mirror, and the front windshield inside the car, which were specified by the rectangular frames, as in Fig. 2. The first two sections were used to observe the side and rear of road condition, the third section can observe the traffic information on the road ahead. Besides, we analyzed the accuracy of arithmetic quiz during driving tasks.

According to the eye tracking data obtained by each participant, we calculated the AOI sample percentage and the AOI fixation duration. The AOI sample percentage was calculated by the ratio of the number of sample points in the AOI of three regions to the total number of sample points. The calculation standard of AOI fixation duration is that the participant's sample points are more than ten consecutively located in the AOI during the experiment. AOI sample percentage and AOI fixation duration were used as the measurement standard to evaluate whether the participants were concerned about the regions related to driving safety. Fixation distribution is a quantitative index based on Voronoi diagrams to describe the degree of aggregation of discrete points, which was used to explore the concentration degree of gaze points^45^. The larger the fixation distribution is, the more dispersed the attentional distribution is.

Linear mixed model was taken to analyze the attention mechanism of participants, including three variables: AOI sample percentage, AOI fixation duration and fixation distribution, which was affected by emotion and cognitive load. Fixed effects were set as inter-group difference and external cognitive load interference, random effects were both of them. 20 experiment trails were selected as repeated effects. Besides, we take Cohen’s d as the criterion to reflect the effect size of statistical tests^46^, with Pearson correlation was applied to explore the relationship between scale scores and attention characteristics. The analysis software was SPSS version 23.0. All tests were two-tailed.

The three-layer network used are 64, 40, and 2. We selected Adam optimization algorithm and binary cross-entropy loss function for model training, and the batch size, initial learning rate, learning rate attenuation coefficient, and parameter retention rate of dropout in the full connected layer are set to 2, 0.01, 0.001, and 0.5, respectively. The model was trained on an NVIDIA RTX 2060 GPU, with CUDA 10 and cuDNN v8, in TensorFlow, using the Keras API. In order to prevent the neural network from overfitting, we used data augmentation in addition to Dropout. We divided the eye tracking data into 6 rows and 8 columns based on the screen area, thus obtaining 48 dimensions of data. Combined with 20 dimensions of AOI sample percentage, 20 dimensions of AOI fixation duration, and 20 dimensions of fixation distribution, each person in the training datasets has 108 dimensions in total. The training data were standardized before being fed into the fully connected network, with ten-fold cross validation applied during model training.

## Results

### AOI sample percentage

**Figure 3:**
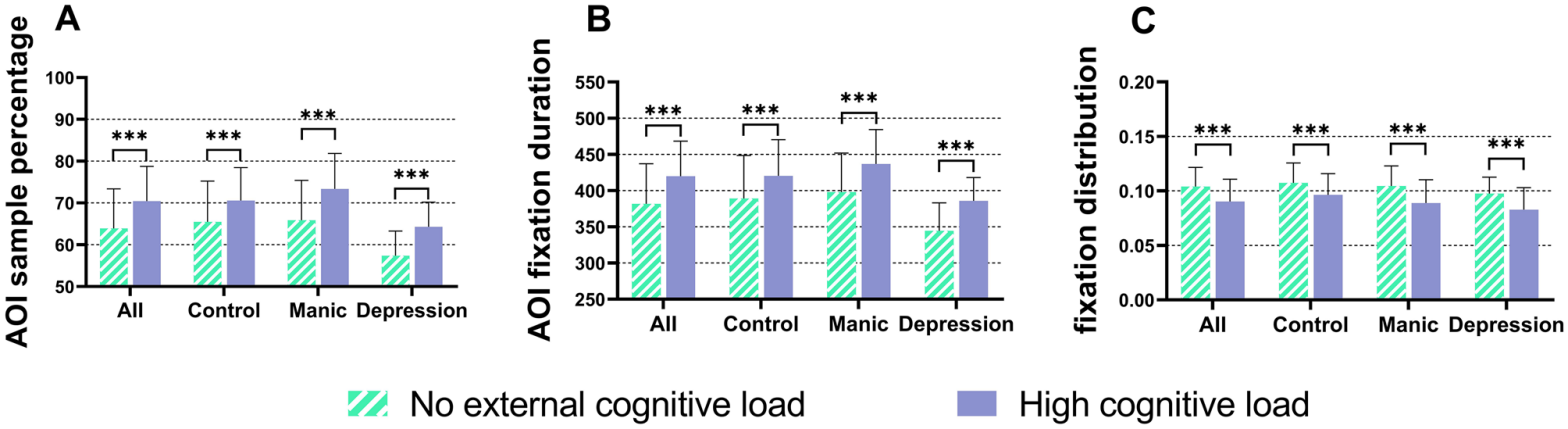
Analysis of significant differences in cognitive load among all subjects and within three groups, ***p < 0.001.

**Figure 4:**
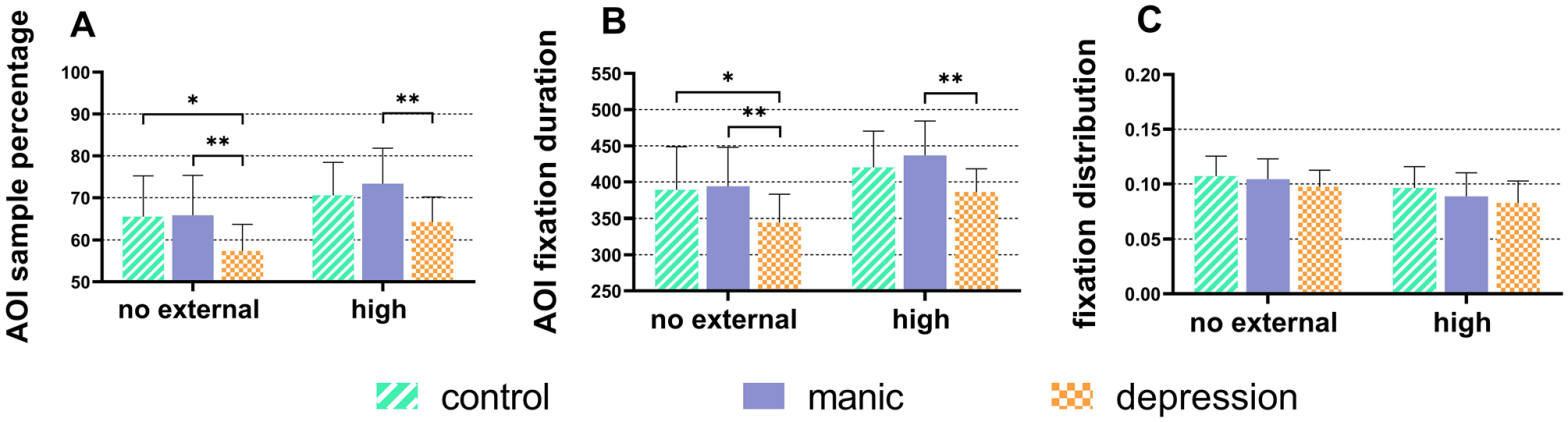
Differences between groups under different cognitive loads, *p < 0.05, **p < 0.01.

As is shown in Fig. 3A, among all the participants, the AOI sample percentage under high cognitive load was significantly higher than that under no external cognitive load, F (1, 48.67) = 90.88, p < 0.001, as well as in the three groups, the data are presented in Table 2. The mean and standard deviation are showed in Figs. 3, 4 and Table 2. Without differentiating the level of cognitive load, the depression group had the lowest AOI sample percentage, reaching 60.84%, the control group and manic group were 68.03% and 69.62% respectively. With the presence of high cognitive load, the AOI sample percentage increased in all groups. Both the manic and depression groups had a greater increase in AOI sample percentage than the control group, increased by 7.52% and 6.91%, respectively, from 65.86% to 73.38% (p < 0.001) and from 57.38% to 64.29% (p < 0.001). The control group increased from 65.50% to 70.56% (p < 0.001), with an increase of 5.06%.

**Table 2:** Means and standard deviations of differences between groups on eye tracking characteristics under different cognitive loads.

Cognitive load	No external cognitive load			High cognitive load		
Group	Control	Manic	Depression	Control	Manic	Depression
AOI sample percentage (%)	65.50 (9.75)	65.86 (9.52)	57.38 (6.35)	70.56 (7.90)	73.38 (8.62)	64.29 (5.91)
AOI fixation duration (s)	389.2 (59.50)	394.0 (53.85)	344.5 (38.6)	420.2 (50.0)	436.8 (47.49)	386.0 (32.0)
Fixation distribution	0.107 (0.018)	0.105 (0.021)	0.097 (0.015)	0.096 (0.020)	0.089 (0.021)	0.083 (0.020)

Without differentiating the level of cognitive load, there were significant differences among the three groups, F (2, 48.96) = 4.48, p = 0.016. The depression group had the lowest AOI sample percentage. The AOI sample percentage in the control group was significantly higher than the depression group (p = 0.029, Cohen’s d = 0.99), as well as between the manic group and depression group (p = 0.005, Cohen’s d = 1.22). Besides, there was no significant difference between the manic group and the control group (p = 0.483, Cohen’s d = 0.18).

Under no external cognitive load, in Fig. 4A, the AOI sample percentage in the manic group was significantly higher than the depression group (p = 0.007, Cohen’s d = 1.05), as well as between the control group and depression group (p = 0.014, Cohen’s d = 0.98). Besides, there was no significant difference between the manic group and the control group (p = 0.881, Cohen’s d = 0.04).

With the presence of high cognitive load, the AOI sample percentage in the manic group was significantly higher than that of the depression group (p = 0.005, Cohen’s d = 1.24), as is shown in Fig. 4A. While there was no significant difference between the manic group and the control group (p = 0.232, Cohen’s d = 0.90), as well as between the depression group and manic group (p = 0.078, Cohen’s d = 0.34).

### AOI fixation duration

Among all the participants, the AOI fixation duration under high cognitive load was significantly higher than that under no external cognitive load, F (1, 48.68) = 85.87, p < 0.001, as well as in three groups, p < 0.001, as is shown in Fig. 3B, the data are presented in Table 2. Without differentiating the level of cognitive load, the depression group had the lowest AOI fixation duration, reaching 365.3 s, while the control group reached 404.7 s and the manic group reached 415.4 s. With the presence of high cognitive load, the AOI fixation duration increased in all groups. Both the manic and depression groups had a greater increase in AOI fixation duration than the control group, increased by 10.9% and 12.0%, respectively, from 394.0 to 436.8 s (p < 0.001) and from 344.5 to 386.0 s (p < 0.001). The control group increased from 389.2 to 420.2 s (p < 0.001), with an increase of 8.0%.

Without differentiating the level of cognitive load, there were significant differences among the three groups, F (2, 48.95) = 4.19, p = 0.021. The participants were divided into the depression group had the lowest AOI fixation duration. The AOI fixation duration in the control group was significantly longer than the depression group (p = 0.043, Cohen’s d = 0.89), as well as between the manic group and depression group (p = 0.006, Cohen’s d = 1.24). Besides, there was no significant difference between the manic group and the control group (p = 0.411, Cohen’s d = 0.21).

Under no external cognitive load, the AOI fixation duration in the manic group was significant longer than that of the depression group (p = 0.008, Cohen’s d = 1.06), as well as between the control group and depression group (p = 0.023, Cohen’s d = 0.89). There was no significant difference between the control group and the manic group (p = 0.708, Cohen’s d = 0.08), as is shown in Fig. 4B.

With the presence of high cognitive load, in Fig. 4B, the control group was significant longer than the depression group (p = 0.105, Cohen’s d = 0.81), as well as between the manic group and depression group (p = 0.008, Cohen’s d = 1.25). While there was no significant difference between the manic group and the control group (p = 0.231, Cohen’s d = 0.34).

Fixation distribution

In Fig. 3C, the fixation distribution under high cognitive load was significantly lower than under no external cognitive load among all the participants, F (1, 48.29) = 82.20, p < 0.001, as well as in three groups, p < 0.001, the data are presented in Table 2. Without differentiating the level of cognitive load, the depression group had the lowest AOI fixation distribution, reaching 0.090, lower than the control group and the mania group, 0.102 and 0.097 respectively. With the presence of high cognitive load, the fixation distribution decreased in all groups. Both the manic and depression groups had a greater decrease in fixation distribution than the control group, decreased by 0.016 and 0.014, respectively, from 0.105 to 0.089 and from 0.097 to 0.083 (p < 0.001). The control group decreased from 0.107 to 0.096 (p < 0.001), with a decrease of 0.011. Without differentiating the level of cognitive load, there were no significant differences among the three groups, F (2, 48.95) = 1.24, p = 0.298.

### Correlation analysis

**Figure 5:**
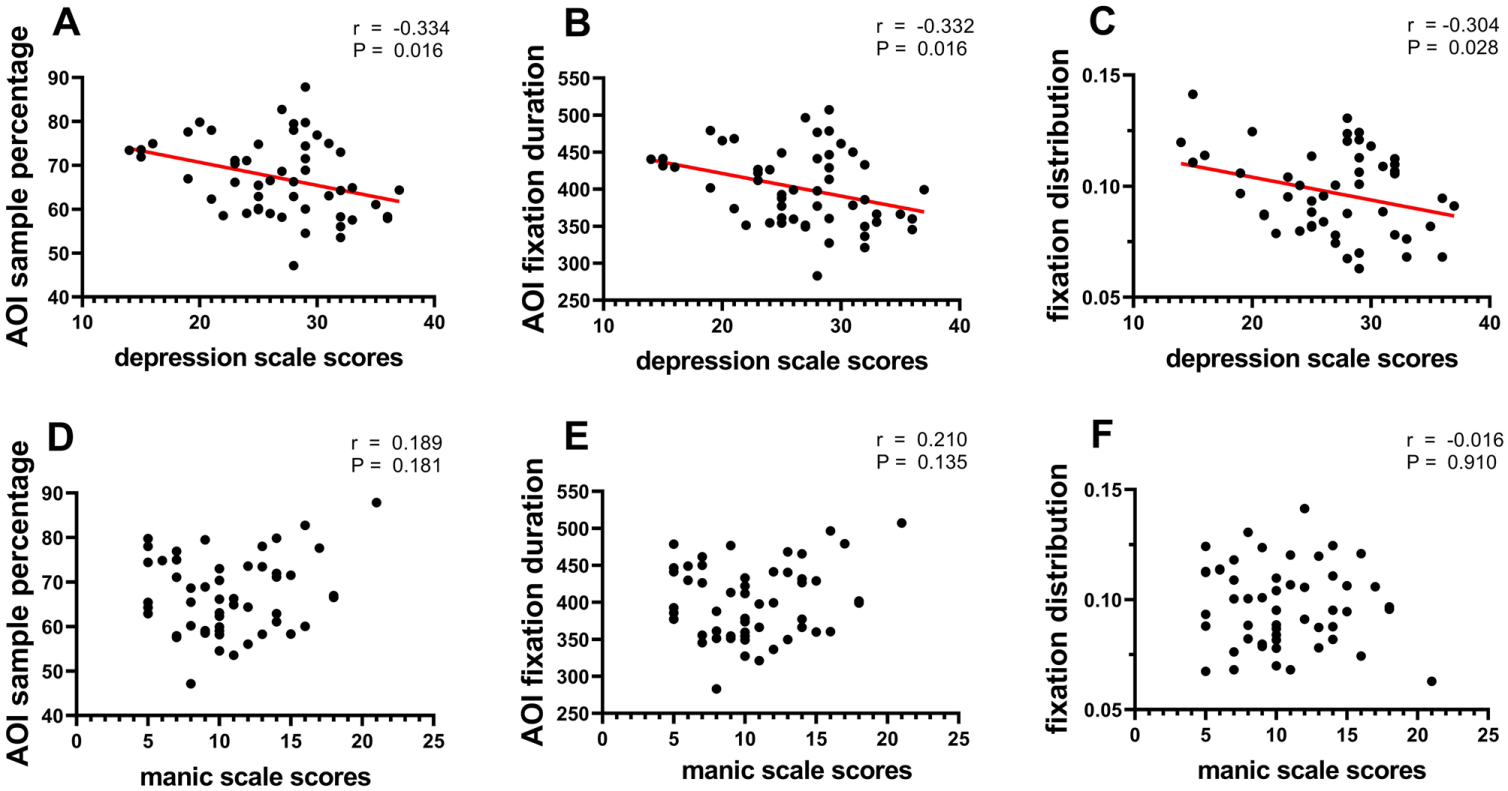
Correlation analysis between scale scores and eye tracking characteristics. The horizontal axis is the scores of the manic scale or the depression scale, and the vertical axis is the eye tracking characteristics including AOI sample percentage, AOI fixation duration and fixation distribution, those with significant correlation are drawn in red lines.

There was a significant negative correlation between eye tracking characteristics and depression scale scores. From Fig. 5, the AOI sample percentage was negatively correlated with depression scale scores (r (52) = − 0.334, p = 0.016, Fig. 5A), as well as between the AOI fixation duration and depression scale scores (r (52) = − 0.332, p = 0.016, Fig. 5B). Besides, fixation distribution was negatively correlated with depression scale scores (r (52) = − 0.304, p = 0.028, Fig. 5C), indicating that the higher participants scored on the depression scale, the more they concentrated on the central area.

In contrast to the depression scale scores, there was no significant correlation between the AOI sample percentage and manic scale scores (r (52) = 0.189, p = 0.181, Fig. 5D), as well as between the AOI fixation duration and manic scale scores (r (52) = 0.210, p = 0.135, Fig. 5E). Besides, there was no correlation between fixation distribution and manic scale scores (r (52) = − 0.016, p = 0.910, Fig. 5F).

### Arithmetic quiz analysis and classification

The results of the arithmetic quiz showed that the accuracy of the control group was highest, reached 63.86%, F (1, 51) = 5.481, p = 0.007. The depression group and the mania group were lower than the control group, 54.55% and 58.48%, respectively. This suggests that the depression group and the manic group may have difficulty multitasking compared to the control group. In our classification tasks, the accuracy of depression group reaches 83.33%. However, the classification accuracy of the mania group could only reach 68.26%. The accuracy of the depression group from the control group is higher than that of the manic group, which is close to 15%.

## Discussion

Consistent with the hypothesis, results confirmed emotion have specific effects on the attention mechanism of driving behavior. In present experiment, we found that the attention mechanism of the depression group was different from that of the control group and the manic group. Under no external cognitive load, the depression group reduced attention to the AOI, which was intuitively reflected in the lower the AOI sample percentage and the shorter AOI fixation duration. Besides, based on the analysis of arithmetic quiz answering in driving tasks, the depression group had the lowest accuracy. To sum up, the attention mechanism of the depression group showed that they paid less attention to driving safety-related information under no external cognitive load, and they were more likely to have difficulties in multitasking in driving behavior when faced with high cognitive load, resulting in negative effects in driving behavior.

Our study support that the depression issues have negative influences on the driving behavior, this is consistent with previous research. Beirness^15^ explored the relationship between depression and driving behavior, high level of depression predisposes individuals to high-risk driving situations, which increased their likelihood of being involved in crashes. Bulmash et al.^47^ conducted the psychomotor disturbance in depression using a driving simulator, patients with depression issues exhibited both slower reactions times and more automobile accidents than controls. Chohedri et al.^48^ combined the Manchester Driving Behavior Questionnaire (MDBQ) and computerized tests to explore the effects of depression on driving behavior. The findings revealed that depression is linked to deficits in a number of cognitive domains, which significantly reduces various aspects of driving behavior. There was no significant difference in eye tracking characteristics between the manic group and control group in our driving tasks.

Crucially, our experiment proved that cognitive load is one of the factors affecting the attention mechanism in driving behavior^49^. Based on the results of fixation distribution, when participants were under high cognitive load in our driving tasks, their attentional distribution was more focused on a small area, tending to pay attention to central area ahead^50^, we call this phenomenon "visual centralization" in driving behavior. One explanation for the "visual centralization" is that drivers may become aware of the increased driving risk, then adopt a prudent strategy, which is reflected in focusing on the road ahead to improve lane maintenance^21^. An alternative explanation for it is that gaze concentration improves tracking response due to enhanced cognitive load^51^.

At the same time, under the condition of high cognitive load during our driving tasks, both AOI sample percentage and AOI fixation duration increased, showing that the participants paid more attention to safety-related information. This is consistent with previous studies that explored the relationship between cognitive load and driving behavior. Engström et al.^49^ exported that with high cognitive load causing drivers to focus their eyes on the road. Hands-free phones could have a protective effect during driving, because the eyes are more fixed on the road ahead under high cognitive load^24,50^. We need to point out that this relationship depends on the analysis of the attention mechanism, the reaction time is another important factor affecting driving safety. The effects of cognitive load on reactions to road conditions and gender differences are factors that need to be taken into account in our future work. Besides, we plan to conduct research on adults in the future to analyze the differences in attention mechanisms between adolescents and adults in the same driving environment.

From the results of correlation analysis, it can be found that in our driving tasks, the higher depression scale scores, the less attention depression group paid to driving safety-related information, which was reflected in the lower AOI sample percentage and the shorter AOI fixation duration. Additionally, we found that as depression scale scores increased, the fixation distribution became smaller, indicating a higher degree of "visual centralization". Based on the correlation analysis of eye tracking characteristics, we may conclude that participants with more serious depression issues, are more likely to have negative effects on driving behavior.

It is worth noting that emotional issues are prevalent in adolescents, which set alarm bells ringing. This paper used the DSM-V assessment scale to analyze the emotional issues of adolescents. Among 52 adolescents we collected, the proportion of adolescents in the depression group and the mania group reached 21% and 42% respectively. Studies have shown that depression and mania are most likely to occur during childhood or adolescence^52,53^. Besides, the number of adolescents in the manic group were twice as many as those in the depression group, indicating that mania has a more serious impact on adolescents than depression in high school. Therefore, we try to combine the eye tracking data with deep learning to detect adolescents with emotional issues. During our experiment, we quantified the attention mechanism of participants. Based on the differences in attention mechanisms between groups, we used the three-layer fully connected neural network to classify the depression group and the manic group from the control group. By optimizing the model parameters, the classification of the depression group reached 83.33%, which was higher than that of mania group. We believe a better detection model can be obtained through the enrichment of psychological experiment paradigms and the improvement of deep learning algorithms.

## Conclusion

Based on our experimental results, depression has a negative impact on driving behavior, which can be concluded from two aspects. On the one hand, participants with depression issues paid less attention to safety-related region under no external cognitive load. On the other hand, they were more prone to have difficulties in multitasking when faced with high cognitive load. During our driving tasks, participants tended to reallocate more attention resources to the central area under high cognitive load, a phenomenon we called "visual centralization" in driving behavior.

## Data Availability

The data that support the findings of this study are available from the corresponding author upon reasonable request.
